# Nurses’ health promotion practices in rural primary health care in Nigeria. A qualitative study

**DOI:** 10.1093/heapro/daae120

**Published:** 2024-09-25

**Authors:** Maria Oluomachi Enebeli, Victoria Saint, Kerstin Hämel

**Affiliations:** Department of Health Services Research and Nursing Science, School of Public Health, Bielefeld University, Universitätsstrasse 25, 33615, Bielefeld, Germany; Department of Population Medicine and Health Services Research, School of Public Health, Bielefeld University, Universitätsstrasse 25, 33615, Bielefeld, Germany; Department of Health Services Research and Nursing Science, School of Public Health, Bielefeld University, Universitätsstrasse 25, 33615, Bielefeld, Germany

**Keywords:** Nigeria, primary health care, health promotion, nurses, rural areas, qualitative study

## Abstract

Nurses play a vital role in providing high-quality primary healthcare and health promotion services. The state of research highlights their often complex operational realities and shows the need for an evidence-based understanding of nurses’ perspectives on health promotion practices, especially in low-resource settings. This study focuses on how community health nurses in rural primary healthcare centers in Nigeria perceive their health promotion role and the opportunities and challenges of, and potential entry points for strengthening, their practice. A sample of 10 nurses from eight rural primary healthcare centers in eight local government areas of Anambra state, Nigeria, was purposively selected. Data were collected via semistructured telephone and written interviews and analyzed by qualitative content analysis using a deductive–inductive approach. Nurses emphasized their commitment to supporting patients and communities to develop skills and take control of their own lives. Nurses described their role as facilitators of behavioral and environmental change, individual and community empowerment facilitators as well as social activists. Factors that enhance the health promotion practice of nurses include adequate skills, sufficient human and material resources and community support and participation. Inhibiting factors included insufficient funding, poor working conditions, staff shortages, high workload, lack of training opportunities and low participation of community members. Overcoming challenges and facilitating health promotion activities in rural communities require bolstering nurses by providing further training opportunities for enhancing their health promotion competencies and creating supportive environments. Future research should focus on how to strengthen nurses’ health promotion efforts through interprofessional and intersectoral collaboration.

**Contribution to Health Promotion**

- Nurses demonstrated a strong commitment to and a dynamic understanding of their health promotion role in changing behavior and empowering and advocating for communities.
- However, they face significant challenges in delivering health promotion services in rural areas, largely due to insufficient funding, staffing and training opportunities.
- Priority areas to strengthen health promotion and primary healthcare practice in Nigeria, as part of efforts toward universal health coverage, include increasing resourcing and training for health promotion activities and addressing health system deficiencies in rural areas to better address structural inequities and social determinants of health.

## BACKGROUND

Health promotion (HP) is one of the core functions of primary health care (PHC) and an essential component of progress toward universal health coverage (UHC) (WHO and UNICEF, 2018). The need to reorient health and health systems from a disease-focused perspective to a multidimensional, socio-ecological concept is well established (WHO, 2018b). This is reflected in the Ottawa Charter for Health Promotion’s three strategies (enable, mediate and advocate) and five priority action areas (develop personal skills, create supportive environments, strengthen community action, reorient health services and build healthy public policy) (WHO, 1986, 2018b). HP enables people to increase control over their health and its determinants, thereby improving health (Kickbusch, 2019; Nutbeam and Muscat, 2021) with individual and community participation playing key roles (WHO, 2008, 2018a). In recent decades, HP has been established as a multidimensional concept requiring application at individual and institutional levels, both in specific settings and as part of multisectoral efforts (e.g. Health in All Policies) (Kickbusch, 2010). Also, competencies and training for HP have been advanced at undergraduate and postgraduate levels, with the professionalization of HP practitioners and specialists as well as the integration of HP in the curricula of nursing and other health professions (Duncan, 2013; Biehl *et al*., 2021). Despite progress, challenges remain in integrating HP as an essential component of health systems, particularly at the PHC level (WHO, 2018b; Biehl *et al*., 2021). Low- and middle-income countries (LMICs) often experience greater challenges than high-income countries in implementing comprehensive PHC with HP components, in particular, countries in sub-Saharan Africa given economic and systems constraints (Starfield, 2009; WHO and UNICEF, 2018).

### Primary health care and health promotion in Nigeria

Nigeria, a country with an approximate population of 216 million has 36 states and 774 local government areas (LGAs) with 53.52% of the population living in urban areas and 46.48% in rural areas (National Bureau of Statistics, 2022). Nigeria operates a three-tier health system with the decentralized organization of PHC at the local government level, secondary health care organized mainly at the state level and tertiary health care at the federal level (FMoH, 2016, 2019). According to the National Primary Health Care Development Agency (NPHCDA, 2015), each LGA is estimated to have a minimum of 10 primary health centers to cover 5,000 persons and above. The country should have at least about 7,740 centers providing health services for children, adults and older people in form of maternal, child, reproductive health, as well as treatment of all ailments and disease prevention (NPHCDA, 2015). PHC services including HP are provided by (local) government-owned public health centers especially in rural areas, as well as private clinics offering promotive, preventive, curative and rehabilitative services (Uzochukwu, 2017; Abubakar *et al*., 2022). The PHC facilities are mostly situated in rural areas to create easy access to health services for the rural population where there are fewer secondary and tertiary health facilities (NPHCDA, 2015). The NPHCDA (2015) coordinates and ensures the provision of minimum quality standards for PHC services. However, PHC is generally weak in Nigeria due to unclear structures and delineation of responsibilities among the three health system levels and low health insurance coverage, among other challenges (Adepoju, 2022). Furthermore, the World Bank (2023) report estimated the poverty rate in Nigeria to have reached 38.9%, with about 87 million Nigerians living below the poverty line particularly in rural areas. This partly explains the significant limitations in access to and breadth of PHC, HP services and other basic health and social services, particularly in rural regions (Abdulraheemet al., 2012; Abubakar *et al*., 2022).

Successive national health policies and bills in Nigeria have recognized HP as an essential component of PHC and national health and development goals (FMoH, 2019; Adepoju, 2022). The first and subsequent Nigerian National Health Promotion Policy (NHPP) aimed to strengthen national health system capacity for PHC and HP, by addressing broader determinants of health and galvanizing health actions from individual to population level (FMoH, 2006, 2019). The Government policy’s theme ‘Promoting the Health of Nigerians to Accelerate Socioeconomic Development’ (FMoH, 2016) emphasizes the crucial role of HP. The NHPP consolidates the coordination of HP stakeholders and activities at the LGA level through PHC structures (FMoH, 2019), with HP explicitly listed as one of the essential services provided by nurses and other health workers in PHC centers (NPHCDA, 2015, 2022). Considering that Nigeria is saddled with a heavy burden of communicable and non-communicable diseases coupled with structural issues such as poor sanitation, inadequate attention to key social determinants of health and low levels of health literacy, the concept of HP proves especially valuable (Uzochukwu, 2017; Boutayeb, 2020). Nevertheless, effective implementation of health standards and policies is yet to be realized due to existing challenges such as insufficient PHC services, poor governance, management and infrastructure, and inadequate healthcare financing and human resourcing (FMoH, 2016; Gyuse *et al*., 2018). In the context of addressing these challenges, the role of health workers such as nurses in ensuring the provision of quality, comprehensive and equitable healthcare and HP services at PHC centers was consolidated (NPHCDA, 2015; NMCN, 2022).

### Community health nurses in PHC and health promotion

The significant role of nurses in providing high-quality PHC has been emphasized. Nurses work with patients and communities by strengthening individual, communal and environmental health competencies, thereby enhancing community participation and empowerment (Kemppainen *et al.*, 2013; Iriarte-Roteta *et al*., 2020; Luisi and Hämel, 2021). Globally, community health nurses (CHNs) constitute the main nursing group involved in PHC, particularly in rural areas in LMICs (WHO, 2017; Martin-Misener *et al*., 2020).

In Nigeria, nurses in public PHC centers, most of whom specialize in community health nursing (NMCN, 2022), are the second largest workforce group after community health extension workers (NPHCDA, 2015; Uzochukwu, 2017). In PHC centers, CHNs play a vital role in promoting, improving and strengthening people’s health. Nurses provide services including: health education and promotion; counseling; home visits and community outreach; oral and mental health; maternal, newborn and child care; family planning; immunization; curative care; referrals; as well as monitoring and supervision (Whitehead, 2010, 2018; NPHCDA, 2015). CHNs are specifically trained to be integrated into communities in which they work, in principle facilitating their greater involvement in the lives of individuals and communities (Emelonye *et al.*, 2016; WHO, 2017; Abdullahi *et al.*, 2019). In reality, significant challenges exist. Studies from LMICs indicate that the promotion of healthy lifestyles is far from integrated in routine nursing function due to barriers in the implementation (Altamimi *et al*., 2016; Jaeger *et al*., 2018; Melariri *et al*., 2021). Studies from high-income countries also show that nurses face significant barriers in HP practice, in particular, due to insufficient human and material resources (Kemppainen *et al.*, 2013; Fonken *et al.*, 2020). Evidence also indicates that rural areas are more severely impacted by such challenges (Roden *et al*., 2016; O’Sullivan *et al*., 2021). The pattern is similar in Nigeria, where nurses and other healthcare professionals are often insufficiently equipped to implement effective HP practices (Samson-Akpan *et al*., 2012, 2013; Uchendu *et al*., 2020), despite its emphasis on the NHPP and national health policies (FMoH, 2019). Further research is needed to understand the specific progress and challenges for advancing HP in rural areas, and in particular to capture the perspectives of nurses given their central role in PHC centers.

## METHODS

### Study aim and design

This study aims to investigate how nurses perceive their roles in HP implementation in their daily activities in rural PHC centers and to identify factors that facilitate or hinder nurses’ HP practice. A qualitative design was employed based on semistructured oral and written interviews (Flick *et al*., 2015). Data were analyzed with qualitative content analysis using a deductive–inductive approach (Kuckartz and Rädiker, 2022). The study was conducted in accordance with the ‘Standards for Reporting Qualitative Research’ (SRQR) guideline (O’Brien *et al*., 2014). The findings can provide insights to guide efforts to implement, strengthen and evaluate HP in rural PHC in Nigeria.

### Setting: Rural PHC centers in Anambra state

The study was conducted in PHC centers in Anambra state. With a population of approximately 5.5 million (> 60% in major cities, < 40% in rural areas) (National Bureau of Statistics, 2020), Anambra is the second most industrialized state in Nigeria after the Lagos metropolitan region and shows strong economic growth (Ndulue and Ayadiuno, 2021). It has several hospitals in urban and suburban cities and at least one PHC center in each of the 326 political administrative wards, mostly in rural communities across 21 LGAs (Government of Anambra state, 2022). Each PHC center covers a minimum of 5,000 people and includes a cadre of personnel as specified in NPHCDA (2015) standards. Anambra is one of several urbanized states in Nigeria that have established their own PHC agency, but despite efforts, successful implementation of the NHPP and national health policy standards and policies has yet to be achieved (ASHIA, 2022).

### Interview guideline

Based on expert interview method (Meuser and Nagel, 2009), the interview guideline was developed to explore CHNs operational and contextual knowledge on the study topic. The guideline’s open-ended questions were developed around the Ottawa Charter’s (WHO, 1986) three HP strategies and five priority action areas. The guideline is divided into five themes:

(1) Meaning and importance of HP;
(2) Support of patient information, education and HP skills development;
(3) Speaking/advocating for patient interests regarding HP;
(4) Contribution of other actors such as management, community and government in supporting nurses to fulfill their HP role; and
(5) Suggestions for improving nursing HP practice in rural communities.

Targeted and narrative questions were used to prompt participants to describe specific situations and experiences in their HP practices.

### Sampling and field access

The study took place between April and September 2022 across eight public PHC centers in eight rural communities of eight LGAs of Anambra state. The first author (M.O.E.) is a nurse with a Master in Public Health and of Nigerian origin which enabled familiarity (Flick, 2018) with the Nigerian health and PHC system. She has no prior relationship with any study participants. The co-authors are of Australian (V.S.) and German (K.H.) origin and supervised the study conceptualization and implementation as part of the first author’s master thesis. All authors live in Germany and could not be in Nigeria for participant recruitment due to political unrest and safety concern during data collection. Convenience sampling (Bornstein *et al*., 2013) and field access were supported by a local contact person (so-called intermediary), who is a personal acquaintance of the first author and a nurse working in an urban hospital in Nigeria. Inclusion criteria were: (a) CHNs with a minimum of 3 years education and qualification as a registered nurse or BSc; (b) minimum of 2 years working experience in PHC centers in rural areas in Anambra state; and (c) good command of English. English is the official language in Nigeria and both nursing education and government documents. The intermediary approached the management of target PHC centers, informed them about the study and asked them to forward information to CHNs meeting inclusion criteria. The intermediary reported to the first author who subsequently contacted potential interview partners via telephone and email. Potential participants received verbal and written information about the study. A total of 10 nurses from different PHC centers were selected to ensure diversity. Due to data saturation (Flick *et al*., 2015), no further sampling was conducted.

### Data collection

Data were collected from ending of June to middle of July 2022. Given the unstable internet connection in rural Nigeria, it was originally planned that the intermediary would accompany nurses to the closest city with better internet access for an interview via video conferencing. Due to the tense security situation in Nigeria at that time, this was waived for the protection of interviewees, who could instead choose between telephone or written interviews (Trier-Bieniek, 2012; Schiek, 2014, 2022). These modes of interview have specific advantages in terms of enabling access to otherwise hard-to-reach participants (Drabble *et al.*, 2016). In the face of insecurity challenges in Nigeria during the research period, telephone and written interviews ensured safety, flexibility, as well as a sense of security and anonymity for both participants and the interviewer (Drabble *et al.*, 2016).

**Fig. 1:**
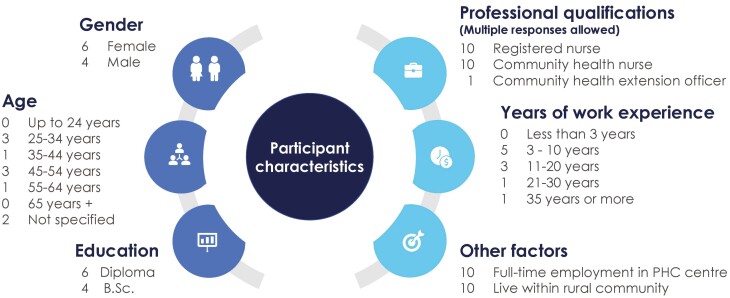
Summary of participants characteristics.

Ten nurses from different PHC centers participated of whom five were interviewed by telephone and five provided written answers. The sample is heterogeneous in terms of gender, age and experience but professional training is relatively similar (Figure 1).

### Data analysis

Telephone interviews lasted from 60 to 120 min (90 min on average) and written interviews encompassed 3–11 pages of text. Recorded interviews were transcribed verbatim and pseudonymized (Dresing and Pehl, 2015); written interviews were also pseudonymized where they allowed inferences about participants.

The data evaluation was based on qualitative content analysis according to Kuckartz and Rädiker (2022). We deductively established five main categories drawing on the interview guide themes. In addition, interview materials were thoroughly read several times and initial notes were made to adapt and further refine these categories; in this step, notes were also used to draft ideas for subcategories which were further developed by inductive coding. In this process, codes were subsumed and adjusted for coherence and consolidation several times. The first author conducted data categorization and coding in consultation with the last author and two master students to ensure consensus and minimize ambiguities and inconsistencies. In conclusion, five main categories and 12 subcategories were identified for the master thesis. However, three relevant categories were selected for this article because they align with the research aim to explore nurses’ perception on their HP practices in rural PHC centers. All categories were subjected to in-depth interpretation by the authorship team.

### Ethics

The Ethics Committee of Bielefeld University approved the study (EUB 2022-125). Data were processed on a network drive in accordance with data protection regulations.

## RESULTS

Participating nurses all indicated that HP is an important, valuable and integral part of their daily practice, which contributes to patient and villager welfare and quality of life. Three main categories were identified in relation to nurses’ HP role and associated facilitating and inhibiting factors (see Table 1).

**Table 1:** Main categories and subcategories

Main category	Subcategories	Definitions
1. Perceived meaning and value of HP as supporting a broader health approach		Describes nurses’ knowledge, understanding and value of HP. This encompass a broader health approach including environmental, socio-cultural and political aspects contributing to health and wellbeing.
2. Nurses’ perceived HP role		Describes the method of action and functions of nurses as well as concrete activities offered by the CHNs in PHC centers to promote population health.
	**Nurse as facilitator of behavioral and environmental change**	Nurses perceive their HP role encompasses strategically informing and counselling people on HP to support behavioral and home environment changes for personal and communal health.
	**Nurse as empowerment facilitator**	Nurses’ person- and community-centered approach is geared toward enabling people to develop skills through individual and collective partnership that will help them to take control of their own health.
	**Nurse as social activist**	Nurses describe their social engagements as means of promoting individual and communal health through advocacy and interventions.
3. Facilitators and inhibitors of nurses’ HP practice		Describes the determinants identified by nurses as facilitating their engagement in and implementation of HP as well as factors inhibiting their HP practice.
	**Systematic and organizational framework**	Nurses emphasize that availability of necessary material and human resources as well as adequate infrastructure and basic amenities influence their practice of HP and thus enhance or inhibit the integration of HP in their daily activities.
	**Community support and participation**	Nurses largely indicate positive material and physical assistance from the community as well as participation expressed in partnership, cooperation and collaboration, which enhances fulfillment of their HP role.
	**Nurses’ competence and feasibility aspects**	Nurses perceive that opportunities for skills acquisition and strengthening their competencies influence their attitude, and availability in rendering HP activities.

### Perceived meaning and value of HP as supporting a broader health approach

All nurses emphasized that HP is of vital significance for the population wellbeing and welfare. It is perceived to enhance autonomy at individual and community levels, and ‘*go beyond* health *care’* (P5_TI_56) (quotes with the acronym TI indicate telephone-based interview) to encompass environmental, socio-cultural and political dimensions. Nurses link their HP activities to their daily nursing routines. Despite emphasizing a broader health approach, the HP activities nurses reported appear to be more preventive than promotive, with a predominant focus on ‘*reducing the increase of infections and diseases’ (P10-WI_26-28)* (quotes with the acronym WI indicates written interview), especially through health education and behavior change.

### Nurses’ perceived HP role

#### Nurse as facilitator of behavioral and environmental change

Firstly, nurses emphasized their focus on sensitizing people to make healthy choices, which is realized through effecting change in their behaviors and home environment.

*This includes change from bad to good like stopping smoking or excessive alcohol […]. Environmental change: cleanliness is next to godliness. Good environment can bring healthy people and when the environment is good, there can be change in lifestyle. So we do advise, encourage and motivate them to change. (P6-WI_45-53)*

These behavioral and home environment changes were seen to be paramount in the rural setting where people experience difficulties in accessing basic amenities like clean water, electricity, good roads and shops with necessary groceries.

Nurses solicit information about individual and community HP needs during consultations in the PHC centers or community contexts and strategically plan how to tackle needs to support individuals and communities to adopt lifestyle changes. This predominantly centers around counseling, information, awareness raising and education using various communication channels to help ‘*patients better understand health needs, equipping them for important individual health decisions’ (P5-TI_71-72)* to support behavior change.

Home visitations appear to play a vital role in nurses’ efforts to reach out to the community, with all participants reporting that this facilitates access to people, especially target groups who are hard to reach or in remote areas. Nurses’ role as facilitators of behavioral and home environmental change is perceived to be facilitated by their being held in high esteem and seen as role models. *‘A health worker must be a role model to help the villagers emulate them […], They look up to the nurse to learn from healthy living from them’ (P7-WI_122-124).*

#### Nurse as empowerment facilitator

A second HP role described by all nurses is as a facilitator who enables people to take control of their health by planning programs and enhancing participative processes that focus on building individual and collective skills.

*We organize empowerment seminars, invite experts in nutrition, public health and healthy living, art and crafts, tailoring etc. To teach those interested in any kind of health issue and handiwork. The goal of all nursing intervention is to help patients and villagers change in things that promote their health, and adaptive functioning. This is achievable by providing free resources to help patient and villagers make healthy lifestyle decisions and changes. (P3-WI_91-95)*

Nurses emphasized the importance of fostering individual and community empowerment that can be independently actionable with available resources in these rural settings, as opposed to approaches that are not feasible or generate dependence. Nurses encourage people to use alternative materials they can easily access at little or no cost. For instance, if boreholes are not available for clean drinking water, nurses advise boiling stream or rain water to make it safer for drinking or using firewood instead of expensive kerosene stoves. High importance is placed on self-sufficiency, which can lead to ripple effects as community members share what they learn from the CHN about effective resource utilization with other community members.

Nurses emphasized the importance of having acceptance and understanding for people and communities in order to advance empowerment goals, allowing villagers to ‘*express opinion about different health promotion implementation, which includes sharing information, feelings and signs’ (P7-WI_275-277)*. Some nurses stressed that comprehension of HP messages and goals is a prerequisite, and reported often using the local language (Igbo) in dissemination and training to ensure understanding. This particularly *‘benefits members of the community who …don’t speak English and those that are not educated’ (P3-WI_65-71).*

#### Nurse as social activist

Nurses describe their role as social activists who engage in health-promoting advocacy and interventions in and for the community. They described actions to promote community connectedness, support and development that strengthens communal skills and social capacity.

*Also mutual cooperation as a community, like helping one another, we also help in developing individual and communal skills […]. They join hands together to clear the swampy areas […], I mean manpower or labor, with the communal skill of teamwork cooperation are developed through active participation which is then very helpful to the entire community. So I help them develop skills by strengthening all the COMMUNITY ACTION for health and active participation. (P5-TI_134-143)*

Nurses clearly understood the critical role that social determinants of health play in their communities, in terms of both socioeconomic status (education, poverty, etc.) and the lack of access to health and other essential services. In the above quote, mobilizing the community to clear swampy areas, rather than wait for improved water, sanitation and hygiene infrastructure. Nurses describe that due to delays in rural areas to receiving government resources and an overall lack of basic infrastructure and amenities, they have shifted to consulting, advocating, mediating, negotiating and partnering with community stakeholders to identify solutions and mobilize resources for HP practice:

*We have to discuss with them, involve them while planning so that they can help and we can work well and carry out our planned program. (P9-TI_171-173)*

Stakeholders include traditional and community rulers, chiefs and elders, church leaders, philanthropists and non-profit organizations. Nurses collaborate with them to provide resources for HP practice rather than waiting for large investments from government to improve social and structural conditions. Six nurses emphasized actively seeking financial and material resources, especially to support people facing barriers in accessing health services, including those living in poverty and lacking health insurance with the inability to pay out of pocket for health services.

### Facilitators and inhibitors of nurses’ HP practice

#### Systematic and organizational framework

All nurses recognized the importance of a supportive environment conducive to HP practice in rural PHC centers, noting significant structural and health system challenges and emphasizing that responsibility for this sits with management and the government.

While some nurses acknowledged support from their management, four nurses specifically noted insufficient managerial and collegial support as well as widespread staff shortages as inhibiting their individual and collective HP practice. While nurses described that *‘collaboration and team work in the health center’ (P10-WI_83-85)*, mutual understanding and good communication are crucial in planning and organizing HP programs, they emphasized that these are lacking.

Areas for improvement highlighted by nurses included recognition from management, seamless communication, affirmation, shared decision-making and involvement in planning.

Nurses were more ambivalent about governmental support to fulfill HP’s roles. While only three nurses cited positive examples, other descriptions were of dissatisfaction:

*We don’t feel the impact of government […] that is supposed to provide us with a car and the equipment [Igbo language], but they don’t do it, […] they don’t provide enough. (P2-TI_431-436)*

Nurses stressed the government’s role in steering and monitoring the implementation of health and HP policies to ensure appropriate HP practice. They emphasized the disconnect between HP policy and its poor implementation, evaluation and funding, particularly in rural areas. One nurse suggested that the government should develop additional HP guidelines tailored to rural contexts, which could enhance the sense of having a comprehensive support system appropriate for their working conditions.

All nurses complained of insufficient resourcing and perceive that within the health system overall *‘mostly neglected are the primary health centers in rural areas’ (P7-WI_167-169).* Poor infrastructure and substandard equipment result in poor working conditions and environments, exacerbated by wider challenges in rural areas, such as unreliable electricity, poor roads making some remote areas inaccessible and poor housing and sanitation. These challenges constitute a primary deterrent for nurses to practice HP and have a considerable negative impact on population health.

Nurses emphasized the need for better management and for the government to ‘*make available, on time, and enough, the basic materials and resources we need to promote the health of our patients and the villagers’ (P4-TI_346-347).* This is essential to enable more effective integration of quality HP in nurses and to foster individual and collective job satisfaction and wellbeing.

#### Community support and participation

Participants reported positive community attitudes and strong individual and community commitment and contributions toward the realization and fulfillment of their HP role.

Support was particularly instrumental from traditional rulers, wealthy community indigenes and religious and non-governmental organizations. Half of the nurses also mentioned support from youth, especially in providing manual labor or assistance. All nurses value community support, however, some expressed concerns about saddling communities with responsibilities meant for the government, which is also reportedly a deterrent for some community members.

Living within the community is reported to enhance the access nurses have to community members, promote the development of mutual relationships and facilitate their availability and readiness to render HP activities. Nevertheless, nurses complained that poor living conditions endanger their health and safety.

Active community participation is reported by all nurses to increase HP program success and efficacy, and outreach by nurses can increase patient and villager participation. This participation strongly contributes to nurses’ job satisfaction.

*Something I noticed since these three years I have been working in this village is that they love to work with us. They really participate in the activities and that is very encouraging and motivating for us, for me as a nurse. That is also one of the reasons why I like community nursing very much. (P5-TI_162-165)*

Community members’ participation in HP activities is reportedly influenced by a range of factors including age, health status, financial status, poverty, health insurance coverage, occupation, values, religious and cultural beliefs as well as interest and willingness to engage. Older patients and villagers are more often involved, which nurses attributed to them having more health conditions and exposure to different health issues. Women generally had higher participation than men and were therefore targeted more. Youth support nurses in organizing HP programs but participate less in actual activities. While nurses understood the challenges of competing commitments, they stressed that poor participation inhibits HP practice and outcomes and so they persist in trying to motivate and encourage participation despite challenges.

#### Nurses’ competence and feasibility aspects

Nurses identified a comprehensive profile of individual skills and environmental opportunities as essential for integrating HP in their daily work.

*We had one of such seminars just recently, […] after these seminars we in turn disseminate the current information to our patients in a way they can understand it. (P3-WI_108-113)*

While nurses value training opportunities and emphasize their readiness to participate, the majority expressed dissatisfaction with insufficient training opportunities, limiting their competencies.

Lack of time was another critical aspect hindering nurses’ ability to organize HP activities in PHC centers or villages and fulfil HP roles. They reported a range of competing responsibilities further aggravated in rural areas where nurses face additional demands, including to supplement or compensation in medical, organizational, mediation, advocacy and social responsibilities and roles.

Nurses emphasized the need for stronger prioritization and more opportunities for skills acquisition and strengthening the individual and collective methodological, communicative and social competencies of front-line health workers. They also called for a reduction of heavy workloads and the provision of safe and supportive working and living conditions. These measures are seen as prerequisites for nursing HP practice and opportunities to facilitate nurses’ professional practice more broadly. Nurses noted that improved HP practice would enhance patients’ and villagers’ participation in HP activities and thus foster their wellbeing, welfare and quality of life.

## DISCUSSION

This study explored Nigerian nurses’ perspectives and experiences related to their HP practices in PHC centers in Nigerian rural areas. The findings improve understanding of this important health workforce group and HP practice in rural PHC centers and point to priority areas to address these central pillars of progress toward UHC in Nigeria. Overall, our findings highlight that nurses have a relatively holistic understanding of their HP role and the structural and social determinants underpinning population health and wellbeing, but the scope of their HP efforts is significantly hamstrung by core health system challenges and poor working and living conditions for them and the communities they serve.

Our study reveals three main components of nurses’ HP role, being facilitators of behavioral and environmental change, empowerment facilitators and social activists. The emphasis on sensitizing people to make healthy choices and take control of their own health, in particular through information sharing, awareness raising and capacity building initiatives aligns with descriptions of the HP role of nurses in other studies from Nigeria and internationally (Kemppainen *et al.*, 2013; Samson-Akpan *et al.*, 2013; Altamimi *et al.*, 2016; Keele, 2019). The interviewees stressed the importance of strategically planning interventions, taking account of individual and communal needs, circumstances and resources. This points to the unique combination of professional, methodological and social competencies in discerning situations and offering appropriate solutions (Okoli *et al*., 2016; WHO, 2017; Amin *et al*., 2020).

Despite aspiring toward a broader approach and holistic HP vision, nurses predominantly described preventive rather than promotive activities and strategies to educate or teach individuals and communities. Previous studies also report nurses’ tendency to focus on educative and preventive medical, rather than promotive, measures (Samson-Akpan *et al*., 2012; Uchendu *et al*., 2020). The NHPP recognizes that Nigeria is saddled with a double burden of communicable and non-communicable diseases (FMoH, 2016, 2019). This double burden seen in many LMICs might contribute to a dominant emphasis on protective and preventive measures (Howze *et al*., 2009; Boutayeb, 2020), i.e. in high-burden, low-resource contexts, a salutogenic and health promotive—rather than disease focused—approach might be especially hard to realize (WHO, 2018b). A deep pragmatism also seems to underpin the interviewed nurses’ focus on supporting improvements for healthier behaviors and home environments using local resources, rather than trying to improve upstream structural and social determinants in the absence of sufficient political will and government support and resourcing.

Whitehead (2018) suggests that many health practitioners, including nurses, believe they are delivering HP when actually engaging in health education, the latter being one narrower component of the more comprehensive HP concept. An educational strategy or approach may lead to more hierarchical methods, manifested in telling people what to do but missing other key HP components such as mutual dialogue and empowerment (Okoli *et al*., 2016; Melariri *et al*., 2021). On the other hand, the nurses interviewed did emphasize the integral role of partnership, collaboration and shared decision-making and enhancing patient autonomy in their health education approach. In our opinion and in the viewpoint of other authors like Teijlingen and colleagues (2021), future research is needed to clarify how health professionals could and have managed to overcome narrower or ‘conventional’ health education strategies by using them as a subset of tools for HP.

Social responsibility is conceptualized by WHO (2017) as a core component of community health nursing. A previous study from Nigeria reported that nurses insufficiently act as social activists because they appeared not to recognize the connection between social policy for health and HP (Samson-Akpan *et al*., 2013). In our study, nurses explicitly embraced their role as social activists, specifically in terms of strengthening community connectedness and development, and individual and collective skills and capacities. Greater health system challenges and resource limitations in rural areas may be one explanatory factor driving nurses to engage more in social actions to help underserved populations (Uzochukwu, 2017; Abubakar *et al*., 2022) or to mobilize support for improving HP practices (Roden *et al*., 2016).

The structural and organizational framework, community participation and support as well as their own professional competencies were key factors identified by nurses as facilitating or inhibiting their HP practice. The most commonly and strongly emphasized facilitating factor was community support as similarly identified by previous studies focused on nursing HP practice and PHC workforce in rural areas (Okoli *et al*., 2016; Jaeger *et al*., 2018; Fonken *et al*., 2020). This result seems to support the suggestion that community participation may be more prevalent and indispensable in rural compared to urban and suburban contexts, underpinned by inequalities in resourcing (Abdulraheem *et al*., 2012; Roden *et al*., 2016). Scholars have stressed that community support cannot be taken for granted to plug gaps in public services (Heusinger *et al*., 2017). Rather, supportive structures for community engagement are essential even more so in the light of accelerating urbanization and demographic transition in Nigeria (Okoli *et al*., 2016; Adepoju, 2022).

Inhibiting factors for nurses’ HP practice included a weak or insufficient support system; insufficient human, financial and material resources; heavy workload and lack of time; poor working and living conditions; as well as insufficient training opportunities for skills and competence acquisition. This corresponds with other recent studies with nurses, physicians and other health workers in Nigeria (Onah *et al*., 2022; Okoroafor *et al*., 2023). The challenges were attributed to insufficient investment in the health system and the neglect of rural areas in its organization, which are also well recognized in existing literature (Ebuehi and Campbell, 2011; Gyuse *et al*., 2018). The National Bureau of Statistics (2020) notes that the Nigerian GDP has been inconsistent showing specifically a decline in health expenditure as a share of GDP since 2003. The PHC sector is the most severely impacted of Nigeria’s three-tier health system (Uzochukwu, 2017). A pattern of poor investment in health and poorer access to health services in rural areas, particularly in LMIC settings, is noted in the international literature (Kafczyk and Hämel, 2021; O’Sullivan *et al*., 2021). We join the call, including from Nigerian nurses interviewed in this study, for more efforts to prioritize investments in health and health-related determinants and address inequities for rural populations, including specifically to better train, equip and support nurses in their PHC and HP practice as well as improve the working conditions and human and material resources (Okoroafor *et al*., 2023). This requires the government and the Ministry of Health to provide the necessary systematic, structural, organizational and infrastructural frameworks as well as systems to ensure efficacious implementation and monitoring of HP policies (Abubakar *et al*., 2022; Adepoju, 2022). These strategies will also foster equitable UHC as prioritized in a new Nigerian initiative and compact for health sector renewal and investment announced in December 2023 (WHO, 2023), as well as strengthen HP implementation that also addresses underlying social determinants of health (Kickbusch, 2007, 2019; WHO, 2017; Iriarte-Roteta, *et al*., 2020) especially for CHNs in rural PHC centers.

### Study limitations

While the first author (M.O.E.) is a Nigerian nurse familiar with the research context, all three researchers were residing in Germany during this study. Differences in the conceptualization and understanding about nursing, HP practice and the wider health system in Nigeria needed to be bridged. The recruitment of participants was based on convenience sampling, which may introduce a selection bias. Logistical challenges, including insufficient electricity and internet in rural areas to sustain video conferencing, and safety concerns due to conflict and unrest, also impacted the study. Telephone and written interviews were therefore adopted, which impacted the ability of the interviewer to observe nonverbal cues and responses, and capture contextual factors about the participants’ work environments and social reality. The possibility of social desirability bias in participant answers can also not be ruled out. We tried to balance risks by inviting the nurses to give comprehensive narrations about specific situations in their everyday work and by creating a confidential space in which the interviewees could openly share their stories (Bergen and Labonté, 2020). Further research is needed to build a more complete picture of the perspectives, state of affairs and opportunities and challenges for HP and PHC in a health system and a country as large and diverse as Nigeria. This should include more in-person and observational studies, with nurses and other cadres of health professions. In this study, the nurses made only little reference to interprofessional collaboration for developing HP activities. Future research should explore the significance of interprofessional and intersectoral collaboration for strengthening HP closer. In addition, the perspectives of community members, including participants with little or no command of English, should be investigated.

## CONCLUSIONS

Nurses contribute significantly to improving the health and wellbeing of people in Nigerian rural communities by integrating HP in their daily activities despite significant challenges such as insufficient funding, staffing and training opportunities. Strengthening CHNs in fulfilling their HP role requires creating supportive environments, particularly for CHNs in PHC centers in rural areas, and providing further training opportunities for acquiring HP skills. Future research should focus on strengthening HP practice of nurses through interprofessional and intersectoral collaboration. National HP and health policies need to be reviewed from this perspective to assess entry points for practical improvement, investment and adequate funding at the personnel, organizational and institutional levels. While this study focuses on Nigeria, the findings presented here can also inform improvement on policies and practices as well as future research in other LMICs with limited resources for health and HP practices.

## Data Availability

Given the potentially disclosive nature of entire interview transcripts and written answers, they will not be made freely publicly available. They are deposited at Bielefeld University and reasonable requests for secure research access will be considered. Please contact: data-access-dept.nursing@uni-bielefeld.de.
